# Safety and Transcriptome Analysis of Live Attenuated *Brucella* Vaccine Strain S2 on Non-pregnant *Cynomolgus* Monkeys Without Abortive Effect on Pregnant *Cynomolgus* Monkeys

**DOI:** 10.3389/fvets.2021.641022

**Published:** 2021-03-09

**Authors:** Shijing Sun, Hui Jiang, Qiaoling Li, Yufu Liu, Qiang Gao, Wei Liu, Yuming Qin, Yu Feng, Xiaowei Peng, Guanlong Xu, Qingchun Shen, Xuezheng Fan, Jiabo Ding, Liangquan Zhu

**Affiliations:** ^1^National/OIE Reference Laboratory for Animal Brucellosis, China Institute of Veterinary Drug Control (IVDC), Beijing, China; ^2^Academy of Agriculture and Animal Husbandry Sciences, Hohhot, China

**Keywords:** brucellosis, transcriptome analysis, gene expression, cynomolgus monkey, vaccine

## Abstract

Brucellosis, caused by *Brucella* spp., is an important zoonotic disease leading to enormous economic losses in livestock, posing a great threat to public health worldwide. The live attenuated *Brucella suis (B. suis)* strain S2, a safe and effective vaccine, is widely used in animals in China. However, S2 vaccination in animals may raise debates and concerns in terms of safety to primates, particularly humans. In this study, we used *cynomolgus* monkey as an animal model to evaluate the safety of the S2 vaccine strain on primates. In addition, we performed transcriptome analysis to determine gene expression profiling on *cynomolgus* monkeys immunized with the S2 vaccine. Our results suggested that the S2 vaccine was safe for *cynomolgus* monkeys. The transcriptome analysis identified 663 differentially expressed genes (DEGs), of which 348 were significantly upregulated and 315 were remarkably downregulated. The Gene Ontology (GO) classification and the Kyoto Encyclopedia of Genes and Genomes (KEGG) pathway analysis indicated that these DEGs were involved in various biological processes (BPs), including the chemokine signaling pathway, actin cytoskeleton regulation, the defense response, immune system processing, and the type-I interferon signaling pathway. The molecular functions of the DEGs were mainly comprised of 2'-5'-oligoadenylate synthetase activity, double-stranded RNA binding, and actin-binding. Moreover, the cellular components of these DEGs included integrin complex, myosin II complex, and blood microparticle. Our findings alleviate the concerns over the safety of the S2 vaccine on primates and provide a genetic basis for the response from a mammalian host following vaccination with the S2 vaccine.

## Introduction

Brucellosis, also known as “undulant fever,” “Mediterranean fever,” or “Malta fever,” is a significant zoonotic disease caused by *Brucella* spp. that is almost invariably transmitted by direct or indirect contact with infected animals or their products (1). Brucellosis remains as an important economic and public health problem in many countries and regions across the world (1, 2). Approximately 500,000 cases of human brucellosis are reported every year globally. However, the estimated incidence was believed to range from 5,000,000 to 12,500,000 annually worldwide (3, 4). Surprisingly, in the recent years, human brucellosis incidences dramatically increased in China (5–8). From April 1, 2007 to March 31, 2017, a total of 435,108 cases of human brucellosis were reported in the Mainland China, with an average of 3,626 cases per month and an SD of 1,834 cases (9). A very recent report from 2013 to 2017 showed that the positive rate of the antibody against *Brucella abortus* was 44.2% in 23,381 serum samples collected from dairy cows with the clinical sign of abortion (10). Interestingly, the incidence rate was strongly correlated with the epidemic kinetics of human brucellosis (*r* = 0.806) (10), which is not too surprising as the cases of human infection with *Brucella* mainly occurred through contact with the Brucella-infected animal(s) and/or animal product(s) (11, 12). Consistently, it is generally believed that the prevention and control of human brucellosis can be satisfactorily achieved by control and/or eradication of the disease in animals (2, 11, 12).

Vaccination against brucellosis remains as the most effective control measure for preventing the disease spread in the high-prevalence regions (12). The WHO and the World Organization for Animal Health (OIE) recommended the live attenuated vaccines Rev.1 (smooth type), S19 (smooth type), and RB51 (rough type), to fight against *Brucella melitensis, B. abortus*, and *B. abortus*, respectively. The abovementioned vaccines are currently extensively used to control animal brucellosis in many countries around the world (11, 13, 14). As for human brucellosis, many groups have made great efforts to develop vaccines for use in the human population; however, thus far, no safe and effective vaccine has been successfully developed and licensed for human brucellosis (14).

In China, the major vaccines used for animal brucellosis include *B. melitensis* strain M5, *Brucella suis* strain S2, and *B. abortus* strain A19. The S2 strain is a live, attenuated smooth phenotype strain that was isolated from the fetus of aborted swine by the China Institute of Veterinary Drug Control (IVDC) in 1952, and it was naturally attenuated to *B. suis* vaccine by a serial passage (15). The advantages of the S2 vaccine include, but are not limited to, lower virulence compared to S19, M5, and Rev.1 in a wider target animal species (pig, cattle, sheep, and goat), and by convenient administration *via* the oral route (15, 16). In China, the S2 vaccine used in the recent decades has shown to be robust, efficacious, and safe in the vaccination of animals. Previously, our studies have shown that the S2 vaccine protected mice from challenges faced by heterologous virulent species, including M28 (*B. melitensis*), 2308 (*B. abortus*), and S1330 (*B. suis*), as evidenced by observations which showed that the immunized mice did not develop any clinical sign or tissue damage (17). Nonetheless, the safety of S2 in primates (e.g., humans) has remained elusive. Given that health workers (e.g., veterinarian) might be accidentally exposed to the vaccine during the vaccination of animals (18), there is a necessity to evaluate the safety of the S2 vaccine strain in human beings. To address this problem, in this study, we examined the safety of the S2 vaccine as well as transcriptome analysis using *cynomolgus* monkeys as an alternative animal infection model. Our results suggest that the S2 vaccine strain was safe to *cynomolgus* monkeys, and immunization with S2 resulted in an altered expression of various genes related to the biological function and the molecular activity of the host.

## Materials and Methods

### Ethical Statement

All animals used in this study were kept and handled in compliance with the Experimental Animal Regulation Ordinances defined by the China National Science and Technology Commission. The study was approved by the Animal Ethical Committee of the China Institute of Veterinary Drug Control under permit number, General Requirements for Laboratory Biological Safety of China, GB19489 -2008.

### Animals

Twelve adult *cynomolgus* monkeys, including six pregnant female monkeys (2-month pregnancy, aged 3.5 years and 3.5 kg of weight) and six non-pregnant female monkeys (aged 3 years and 2.5 kg of weight), were purchased from Guangxi Province, China. Upon arrival, all monkeys were serologically tested by the Rose Bengal Plate Agglutination Test (RBPT) (19) and competitive ELISA (Svanova, Switzerland) and were confirmed to be *Brucella*-negative. All animals and all animal experiments were performed in an absolute negative pressure closed area conforming to BSL-3 or ABSL-3 conditions.

### Experiment of Immunization

The lyophilized *Brucella* live S2 vaccine (with an amount of 1.22 × 10^10^ CFU and 80 doses/bottle), purchased from Qilu Animal Health Co. Ltd., China, and stored at −20°C in the fridge of the National/OIE Laboratory for Brucellosis of IVDC for no more than 3 months after their arrival, was used for immunization. One bottle of stock S2 vaccine was diluted in 200 ml of phosphate buffered saline (PBS) (pH7.2) to a final concentration of 5 × 10^9^ CFU/ml. Four pregnant and four non-pregnant monkeys were randomly selected. Each monkey was subcutaneously immunized on the thigh with 1 ml (equivalent to 5 × 10^9^ CFU) of the diluted S2 vaccine, according to the Chinese Veterinary Pharmacopeia (Version 2015) (19). Placebo injection was performed using an identical volume of PBS (pH7.2) on two pregnant and two non-pregnant monkeys as negative controls.

### Monitoring of Animals

All animals were monitored daily for 14 consecutive days post-immunization (p.i.). The immunization spot of the skin on the thigh was examined for any abnormal signs, such as red swelling, ulceration, and local inflammation. In addition, the behaviors and the mental status of all animals were monitored during activities such as walking, eating, drinking, as well as other similar activities. Starting from 3 days prior to immunization, the body temperature of all monkeys, except for the pregnant animals, was measured and recorded each morning and afternoon. The body temperature of the pregnant monkeys was not tested to minimize the influence on pregnancy. All pregnant monkeys were continuously monitored for abortion or any abortion-like signs after immunization until delivery. The clinical observation period of the test lasted for about 9 months till the weaning of the offspring.

### RNA-Sequencing

On day 4 p.i., blood was collected from non-pregnant experimental monkeys. Subsequently, the peripheral blood mononuclear cells (PBMC) were isolated according to the protocol of the manufacturer (Solarbio Life Sciences, Beijing, China). Briefly, Reagents A and D in the kit were gently added into a 15 ml centrifuge tube, resulting in a layered solution. The blood sample with a volume equivalent to that of the layered solution was transferred onto the top of the layered solution and then centrifuged for 25 min at 500 g, resulting in a solution with five visible layers. The solutions in the second and third layers were gently collected and transferred into a new 15 ml centrifuge tube, and then, a 10 ml of wash solution was added and mixed gently. The mixture was centrifuged for 10 min at 250 g, and the supernatant was disposed. The pelleted cells were suspended in a 5 ml wash solution and subjected to centrifugation as above. The cells were again washed three times as above, suspended in a 5 ml wash solution and used for total RNA extraction and purification. The purity, molar concentration, and the integrity of the RNA were determined by Nanodrop (ND 1000 spectrophotometer Thermo Scientific, Wilmington, USA), Qubit 2.0 (Thermo Fisher Scientific, MA, USA), and Aglient 2100 (Agilent Technologies Inc, CA, USA), respectively, to ensure that the samples used for the transcriptomic sequencing were qualified. Subsequently, a cDNA library was constructed using the extracted RNA. Briefly, the eukaryotic mRNA was enriched using magnetic beads and Oligo (dT). A fragmentation buffer was then added to randomly disrupt the mRNA. The first cDNA strand was synthesized utilizing random hexamers as primers and mRNA as a template. The second cDNA strand was synthesized after adding a buffer, dNTPs, RNase H, and a DNA polymerase. The cDNA of each sample was purified by AMPure XP beads (Beckman Coulter, CA, USA) and subjected to end repair, A-tail addition, and adapter sequencing. The fragments with appropriate size were selected by AMPure XP beads. The concentration and size of the fragments inserted in the library were measured using Qubit 2.0 and Agilent 2100, respectively. Finally, PCR enrichment was performed to construct the cDNA library for each sample using the NEBNext Ultra Directional RNA Library Prep Kit for Illumina (New England BioLabs, MA, USA) as recommended by the manufacturer. The accurate concentration of the library was determined by using a real-time PCR. High-throughput sequencing was conducted by the HiSeq X Ten System with a read length of PE150. Sequencing and subsequent bioinformatics analyses were completed at Biomarker Technologies Co., Ltd, Beijing, China.

### Identification of Differentially Expressed Genes (DEGs)

Identification and characterization of DEGs were achieved based on the results of differential expression analysis *via* EBSeq algorithm to filter DEGs with a log-fold expression change (log2FC > 0.585 or < −0.585) using a threshold of false discovery rates (FDR < 0.05), as previously described (17).

### Gene Ontology (GO) Analysis and Kyoto Encyclopedia of Genes and Genomes (KEGG)

#### Pathway Analysis

Comparative transcriptome analyses, employing the GO classification and the KEGG pathway analysis approaches, were performed. The gene products were annotated in terms of a biological function and metabolic pathways. The biological function, signal transduction, and metabolic pathways of the DEGs were analyzed.

#### Quantitative Reverse-Transcription PCR

To validate the difference of gene expression profiling, a quantitative reverse-transcription PCR (qRT-PCR) was performed. A total of 23 DEGs were randomly selected, including 12 upregulated and 11 downregulated genes. The targeted genes and the designed primers used for qRT-PCR are shown in Table 1. Briefly, the mRNAs from samples of six non-pregnant monkeys were extracted and used for the following qRT-PCR system. The reaction mixture was prepared for reverse transcription, including 5 × PrimeScript^TM^ RT Master Mix [containing PrimeScriptRTase, RNase inhibitor, Random Hex Primer, Oligo (dT), and dNTPs] (Takara Bio, Dalian, China) of 2 μl, Total RNA of 5 μl, and sterile RNase-free H_2_O of 3 μl. The mixture was incubated at 37°C in a water bath for 15 min for reverse transcription, followed by incubation at 85°C in a water bath for 5 s for the inactivation of reverse transcriptase. The cDNA was 10-fold diluted using a sterile RNase-free H_2_O and used for PCR amplification. The PCR reaction system consisted of 10 μl 2 × TB Green Premix Ex Taq (Tli RNase H Plus), 0.4 μl 50 × ROX Reference Dye II, and 6 μl sterile RNase-free H_2_O. The mixture was incubated at 95°C for 30 s and then subjected to 40 cycles of incubation at 95°C for 5 s and 60°C for 34 s in the LightCycler 2.0 system (Roche Life Sciences, Basel, Switzerland). To normalize the expression, the β-actin of *cynomolgus* monkey was used as an internal amplification control, for which the forward primer ACTB-1 (5′-CGCCATGGATGATGATATCGC-3′C) and the reverse primer ACTB-2 (5′-ATCCTTCTGACCCATGCCCA-3′A) were used, and the preparation of the same mixture as well as thermocycling were performed.

**Table 1:** Transcriptional level of 23 randomly selected genes by RNA-seq and qRT-PCR.

**Gene**	**Change of regulation**	**Sequence (5'-3')**	**Fragment length (bp)**	**Log**_****2****_ **FC (S2 vaccinated monkeys vs. negative control**		**Annotation**
				**RNA-seq**	**qRT-PCR**	
T-DYSF	Up	GGGAACCGCTACCATCTACG	246	5.1790	4.0874	Dysferlin isoform X1
		GTCGTACAGCTCCACCACAA				
T-OASL	Up	AGACGGCCAACCTGTTAAGG	162	3.2444	1.6045	2'-5'-oligoadenylate synthase-like protein
		TCCGGTGCTCTATGGGATGA				
T-GBP1 (LOC)	Up	TTCCTGTCACCCCTACCCAA	130	3.1717	3.7587	Guanylate-binding protein 1
		TTGATCACTGTACTGCATGCCT				
T-APOL6	Up	ATGACCAAGAATGCTCGCCT	224	3.1460	4.0167	Apolipoprotein L6
		GGCTCAGAGGCCTAACAGTA				
T-GBP2	Up	TGGACCAAACGTTCCAGAGG	234	2.8084	2.7357	Uncharacterized protein LOC105761007
		CCTTCCTTGGCACCTGGTAG				
T-IFIT2	Up	AGAACGCCATTGACCCTCTG	106	2.7773	4.3010	Interferon(IFN)-induced protein with tetratricopeptide repeats 2-like
		TCGCCTTCTTCACGCATCTT				
T-WARS	Up	TGCTGAACGAAGCCTCTGAC	175	2.4982	3.2010	Tryptophan–tRNA ligase
		CAAAAGTGATGGCTTTGACGCA				
T-LOC102143453	Up	AAACTTGGAGCTGTGGTGCT	118	2.4676	5.3050	Guanylate-binding protein 6
		TGGGGGCTGTCCTTTCTAGT				
T-IRF7	Up	CGACTGTGGCACCCAGG	105	2.2691	6.5161	IFN regulatory factor 7 isoform X1
		GTGTGACTGCAGGTATGGCT				
T-APOL2	Up	AGCAGGGGAGTGGTTAATGC	157	2.1390	3.7849	Apolipoprotein L3-like isoform
		CCCCTCTATTCCTCCCCCAA				
T-LITAF	Up	GCGTCTGCAGCAAAGGTAAA	168	2.0562	0.6692	Lipopolysaccharide-induced tumor necrosis factor-alpha factor isoform X1
		CTGTCACAAGCCCCGTAGTT				
T-CTSD	Up	GGCGAGTACATGATCCCCTG	111	1.1492	2.3983	Cathepsin D
		CGACACCTTGAGCGTGTAGT				
T-RPL18A	Down	GCTTCTGGCACACTACGAGA	202	−0.6245	−2.6684	60S ribosomal protein L18a
		TCTCAAACACCTGCCCACAG				
T- RPS13	Down	GAGGTCACTTCCTGCGTGTT	186	−0.6301	−0.1930	Predicted: 40S ribosomal protein S13
		GCTCCTTCACGTCGTCAGAT				
T- FAM102A	Down	GGAGACCCCTGCTTCAAGAC	138	−0.6932	−0.9073	Protein FAM102A
		GGACCCAGTCAAGGAGTTGG				
T- ZFP36L2	Down	ACCAGCAGCTATCAACCACC	205	−0.7100	−3.0286	Zinc finger protein 36, C3H1 type-like 2
		ACCTATGGGCTGAGGGCTAA				
T-CXCR4	Down	GCTGTTGGCTGAAAAGGTGG	204	−0.7495	−1.7388	B-cell CLL/lymphoma 9-like protein isoform X1
		GACAGGATGGCGATACCAGG				
T-GNLY	Down	CTCCCACATCGTGGAGACCTG	237	−0.8416	−3.7190	c-X-C chemokine receptor type 4
		TGTAGGCCTGAGGAAACGCA				
T-LOC102143791	Down	GAGCTCAGGACATTTTCTTCCC	211	−0.9605	−2.6477	Integrin-alpha FG-GAP repeat-containing protein 2
		GAGGCAGGCCTTCGCAAAAA				
T-ICAM2	Down	TTGTGAACTGGGCAGCTGTG	105	−1.0497	−0.2942	Granulysin
		GAAGGGCTGCCCCTGTG				
T-RPL29	Down	AGAGGACGGGTGCAGACA	139	−1.1085	−1.3181	MHC class I heavy chain
		CTCAGGAACTTGGGGTCCAC				
T-BCL9L	Down	GGTATTGAGATCAAGGCGTCCA	225	−1.2431	−2.7988	Intercellular adhesion molecule 2
		TGGAGCACGGACTCAGGAAT				
T-ITFG2	Down	GTCCCGAACATCTCACAGGG	239	−1.4577	−0.5008	60S ribosomal protein L29
		CAGCACCACATCTCGGGTAG				

## Results

### Clinical Assessment

On the day of immunization, very slight swelling was observed at the injection site of the thigh skin of two *cynomolgus* monkeys (data not shown). However, these observatory signs rapidly vanished on the next day p.i. Afterward, no abnormal clinical manifestation was observed on the skin of the immunized monkeys until the end of the experiment, indicating that S2 immunization did not result in deleterious clinical effects at the injection spot of the monkeys.

**Figure 1:**
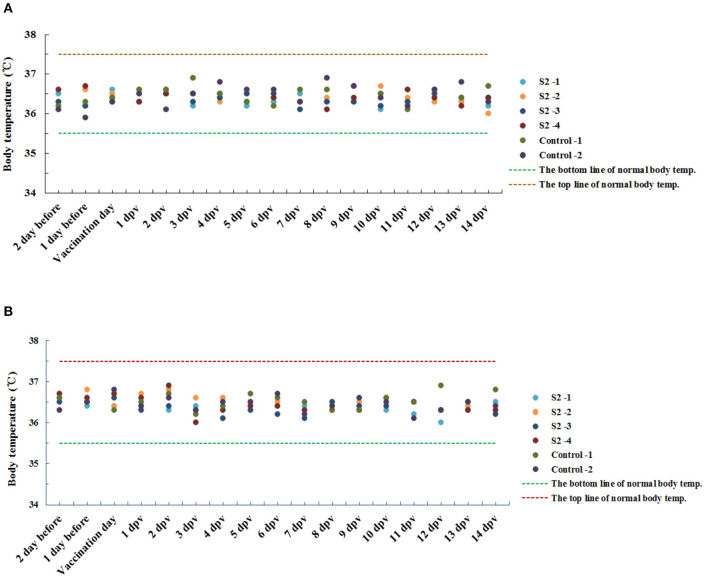
Results of body temperature monitoring of *cynomolgus* monkeys immunized with the S2 vaccine and inoculated with phosphate buffered saline (PBS) (negative control). The body temperature of all non-pregnant *cynomolgus* monkeys, including the animals immunized with the S2 vaccine and those inoculated with PBS, was detected daily during the entire experimental period, starting from 3 days prior to injection till 14 days post-immunization (p.i.). **(A)** Indicated the results of body temperature of all the animals detected in the morning, and **(B)** showed the results obtained in the afternoon. The bottom line of normal body temperature (35.5°C) and the top line of normal body temperature (35.7°C) were indicated.

The body temperature of all non-pregnant *cynomolgus* monkeys was detected daily during the whole experimental period, starting from 3 days before immunization until 14 days p.i. The results indicated that the temperature of all non-pregnant monkeys monitored throughout the entire experimental period fell into a normal level, ranging from 35.5°C to 37.5°C, and no difference was observed between monkeys immunized with S2 and those inoculated with PBS (Figure 1). These results demonstrated that immunization of S2 and PBS did not lead to abnormal change in the body temperature of the non-pregnant monkeys.

The behavior of all monkeys was monitored daily during the whole experimental period. Our results showed that none of the animals exhibited any abnormal behavior (data not shown). No difference was observed between the S2-vaccinated and PBS-inoculated animals in terms of behavior over the whole experimental period, including activities such as drinking and eating. These results suggested that the S2 vaccination did not alter the behavior of any *cynomolgus* monkeys.

#### Monitoring of Abortion

A previous study reported that natural infection of *Brucella* spp. frequently resulted in abortion (1). Therefore, the clinical sign of abortion is an important indicator for the infection of *Brucella* spp. In order to understand whether immunization of the S2 vaccine leads to abortion, we inoculated four pregnant *cynomolgus* monkeys with S2 and then continuously monitored them on a daily basis for abortion till delivery. None of the immunized monkeys got aborted after vaccination. Moreover, the offspring of monkeys did not exhibit abnormal behaviors or clinical signs (data not shown), thus suggesting that the S2 vaccination did not cause abortion in the *cynomolgus* monkeys.

### Transcriptomic Analysis of S2-Vaccinated and PBS-Inoculated *Cynomolgus* Monkeys

**Figure 2:**
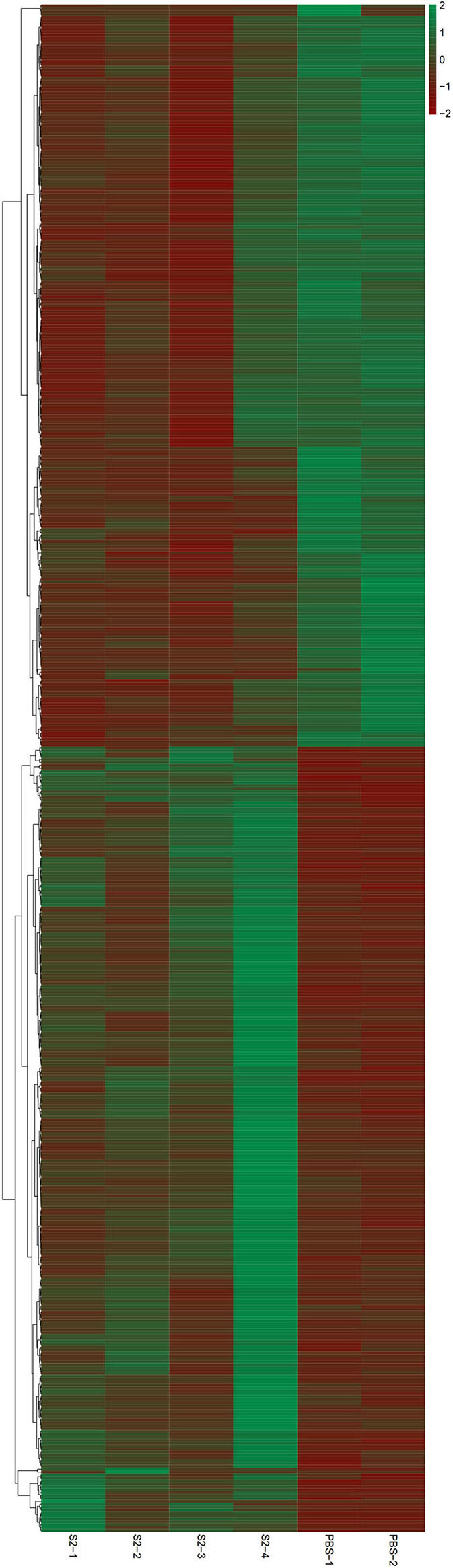
Heatmap for RNA-sequencing (RNA-seq) data. Blood of four *cynomolgus* monkeys immunized with the S2 vaccine and that of two *cynomolgus* monkeys inoculated with PBS (negative control) were collected and used for RNA extraction. A cDNA library was constructed and then transcriptomic RNA-seq was performed. Red and blue pixels indicate upregulated and downregulated genes, respectively. Genes were categorized into different clusters based on the generated dendrogram.

To identify the genetic factors correlating with S2 immunization, changes of the transcriptomic profile of gene expression in *cynomolgus* monkeys immunized with S2 were examined by a transcriptomic analysis. The same analysis was also performed on the negative control samples collected from *cynomolgus* monkeys inoculated with PBS. RNA extracted from the blood of non-pregnant monkeys was used as a template for the construction of a library, followed by a transcriptomic RNA-seq. We did not collect blood from pregnant *cynomolgus* monkeys, considering that the fixation of animal and blood collection may lead to unexpected abortion. Identification and analysis of DEGs were performed based on the results of EBSeq algorithm analyses. On day 4 p.i., transcriptomic sequencing of the mRNA of 15,009 genes derived from the S2-immunized group identified 663 DEGs (log2FC > 0.585 or < −0.585 and FDR < 0.05), compared to the negative control group (in which animals were inoculated with PBS), including 348 significantly upregulated genes and 315 significantly downregulated genes (Figure 2). Among these DEGs, the highest-ranked upregulated genes included DEXH (Asp-Glu-X-His) box polypeptide 58 (DHX58), integrin-alpha 2 (ITGA2), and 2'-5'-oligoadenylate synthetase 2 (OAS2), whereas the lowest downregulated genes comprised of myosin VIIA (MYO7A), Usher syndrome 1C (USH1C), and chemokine (C-X-C motif) receptor 6 (CXCR6) (Figure 2).

### Gene Ontology *Category*

**Figure 3:**
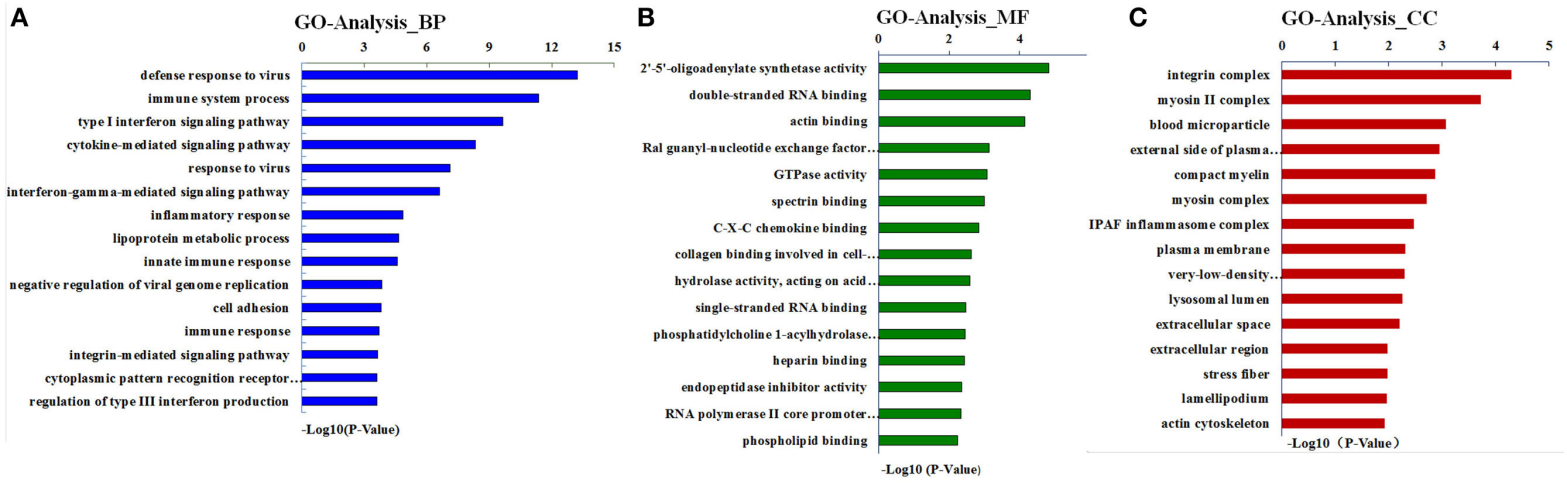
Gene ontology (GO) analysis for differentially expressed genes (DEGs). Results are grouped by three main functional categories, including **(A)** the biological process (BP), **(B)** molecular function (MF), and **(C)** cellular component (CC).

The term GO has three ontologies, including the biological process (BP), molecular function (MF), and cellular component (CC). The sequences of these aforementioned DEGs were analyzed employing a GO analytical approach. Our investigations based on the GO functional enrichment analysis revealed that the 663 DEGs were mainly involved in various BPs, including defense response, immune system processing, and the type-I interferon signaling pathway (Figure 3). The MF of these DEGs included 2'-5'-oligoadenylate synthetase activity, double-stranded RNA (dsRNA) binding, and actin-binding (Figure 3). In addition, these genes were correlated with C, including integrin complex, myosin II complex, and blood microparticle (Figure 3).

### Pathway Analysis

**Figure 4:**
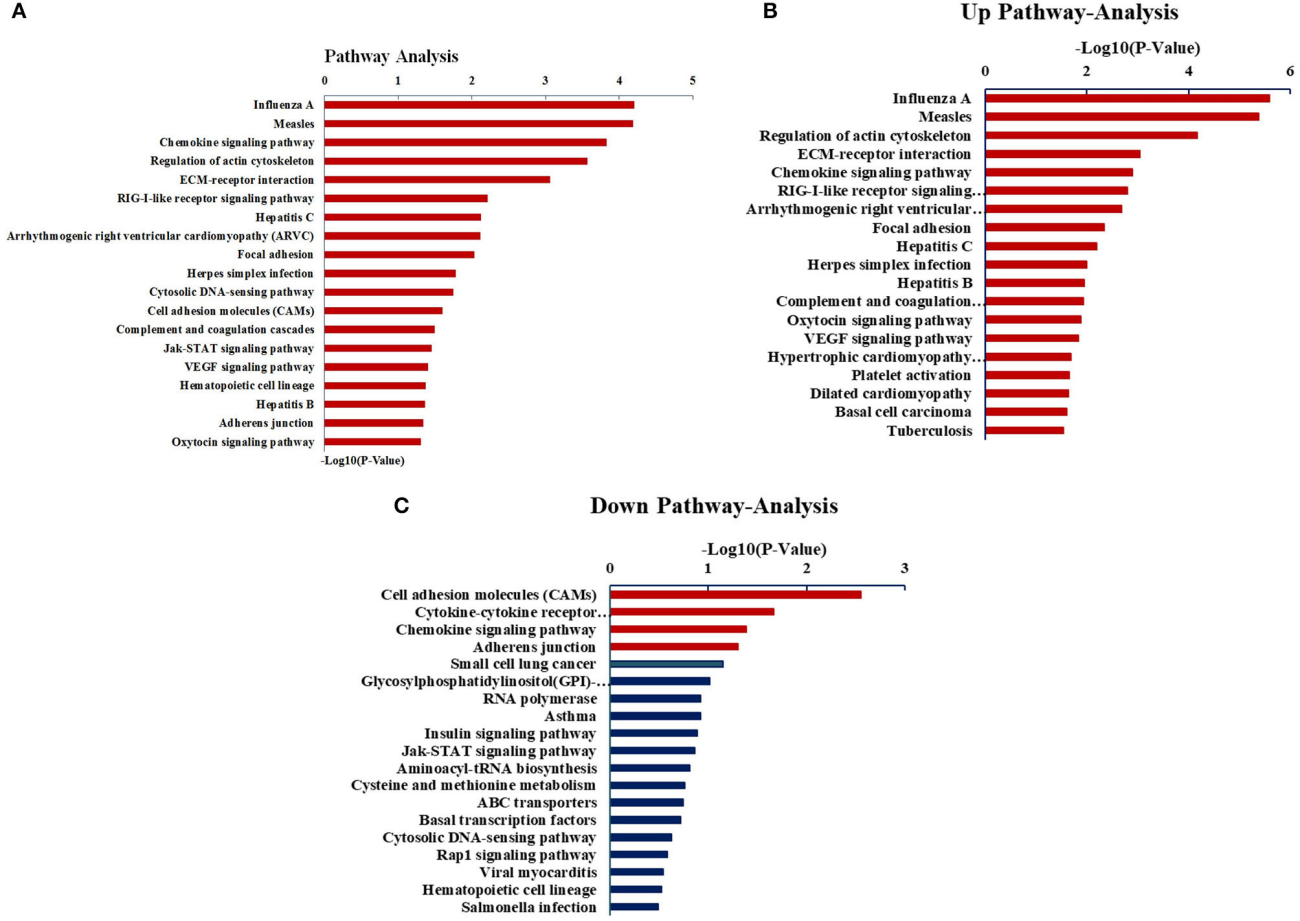
Analysis of DEGs through the KEGG pathway. **(A)** The KEGG pathway analysis of all DEGs. **(B)** The KEGG pathway analysis of upregulated expressed genes. **(C)** The KEGG pathway analysis of downregulated expressed genes.

Next, we sought to analyze the above DEGs *via* the KEGG pathway analysis. As shown in Figure 4, the DEGs were mainly related to the chemokine signaling pathways, signaling pathways after measles virus, and the infection of influenza A virus. Interestingly, the upregulated genes were enriched in pathways relating to the regulation of actin cytoskeleton, as well as the infection by measles virus and influenza A virus, whereas the enriched downregulated genes were relevant to cell adhesion molecules, cytokine–cytokine receptor interaction, and chemokine signaling pathway.

#### Quantitative Reverse-Transcription Polymerase Chain Reaction

To validate the RNA-seq results, a total of 23 DEGs, including 12 significantly upregulated genes and 11 significantly downregulated genes, were randomly selected and quantified by qRT-PCR. The β-actin gene was simultaneously detected and analyzed as an internal control. As outlined in Table 1, the expression levels of the 23 selected genes in the qRT-PCR exhibited a very similar profile to that of the RNA-seq, confirming the consistency of results obtained in the RNA-seq and the qRT-PCR.

## Discussion

Brucellosis is a worldwide zoonotic infectious disease caused by the members of the genus, *Brucellae*, which are circulating in many countries, especially in the Mediterranean basin, Asia, the Middle East, and in the Central and South America (20). Brucellosis, which leads to substantial economic losses in the animal industry, has a significant impact on the health of human populations, as well as has an influence on the structure of the whole society around the world, e.g., public safety, food hygiene, and foreign trade (21, 22). A recent report described the analyses of the species distribution, host lineage profiles, genetic relatedness, and the epidemiological correlation of the *Brucella* species in countries along the Silk Road. The authors concluded that numerous animal reservoirs were a potential reason for the continuous circulation of the brucellosis in the examined countries, while the *B. abortus* strains were dominated in these countries and spreading within the national borders (23). In addition, using descriptive analysis and meta-analysis approaches, Zhou et al. reported very recently that dogs and yaks were the leading reservoirs and emerging hosts for *Brucella* transmission in China (24). Meanwhile, the authors inferred that humans probably get infected along with different kinds of animal species, among which the sheep and goats were likely at the highest risks (24). The aforementioned studies, as well as the ligatures, e.g., a recent report from our collaborators (25), consistently demonstrated the significance of the One Health concept and recommended implementing it to disrupt the *Brucella* transmission chain between animals and humans, as well as to strengthen the close collaboration between the local and public partnerships.

It has been well-documented that vaccination plays a critical role in the prevention and control of brucellosis (11, 14, 17). However, so far, there has been no licensed vaccine for brucellosis in human, and currently, the available animal vaccines may cause huge concern and disease; therefore, they were considered unsuitable for use in human populations (26). Therefore, the search for brucellosis vaccine for humans has continued to raise the interests of the researchers. As far as vaccines against animal brucellosis are concerned, there are currently a variety of vaccines available in the world. The *Brucella* live vaccines, S19, Rev.1, and RB51, which are widely used in different regions of the world, have been demonstrated to effectively offer immune protection against brucellosis (11, 14, 17). Nevertheless, the negative implications of these vaccines, such as the residual virulence in the host, diagnostic interference, and the pathogenic effect to human health, in particular, have remained poorly understood, thus raising doubts and concerns in the implementation of these vaccines in the field conditions (11, 14, 17, 27). For instance, a previous study showed that although the vaccine strain S19 was of low virulence for cattle, the vaccination of pregnant cows resulted in abortion with incidence rate ranging from <1–2.5% under field conditions (12). These concerns and problems prompted us to evaluate the safety of live attenuated *Brucella* vaccine strain in primates. Given that the virulence of the S2 strain was the lowest compared to other *Brucella* vaccine strains (17, 28), we explored it to determine the safety of S2 on *cynomolgus* monkeys.

The S2 vaccine is extensively used in China to control brucellosis in goats, sheep, and cattle. In our previous study, we demonstrated that the S2 vaccine could protect mice from virulent challenge by heterologous strains, including *B. militensis* M28, *B. abortus* 2308, and *B. suis* 1,330 (17). However, the safety of S2 in primates, especially humans, has remained undetermined. The possibility of the infection of S2 vaccine strain in a group of veterinary professionals due to unexpected reasons, such as accident exposure while handling the vaccine, should not be ignored, considering the fact that S2 is a live vaccine. Accordingly, the safety of the S2 vaccine to humans needs to be comprehensively evaluated. In addition, it has been well-documented that cases of *Brucella* infection in human populations were predominantly caused by animals or animal products infected with *Brucella* (11, 12). Thus, prevention, control, and elimination of *Brucella* in animals would be extremely beneficial to the control of human brucellosis. Therefore, in the present study, we sought to determine the safety of the S2 strain to humans using the *cynomolgus* monkey infection model. Our results clearly showed that the S2 vaccine was indeed safe to *cynomolgus* monkeys, as evidenced by observations that all monkeys immunized with the S2 strain neither showed any abnormal behaviors nor suffered abortion. This evidence might alleviate concerns of grass-root veterinarians over the accidental exposure to the S2 vaccine strain when using it to immunize animals in practice. In other words, our data would enhance the confidence of professionals in the use of the S2 vaccine in the field conditions. To the best of our knowledge, this is the first report of evaluation on live attenuated *Brucella* spp. vaccine in *cynomolgus* monkeys, which is an ideal primate species as it is genetically close to human beings. Furthermore, given that the S2 strain was highly attenuated in mice (17, 28) and was safe to *cynomolgus* monkeys as described here, it might be feasible to further attenuate the S2 strain while keeping its immunogenicity unchanged using genetic engineering, thus making it a potential vaccine candidate for use in human populations.

Very recently, a total of 1,205 DEGs were identified between artificially induced rough-mutant *Brucella* strain RM57 and its parent strain, M1981, *via* the transcriptome analysis (29). The goal of this study was to evaluate the safety of the live vaccine S2 in human utilizing *cynomolgus* monkeys as an alternative model, as well as performing the transcriptome analysis of the peripheral blood collected from S2-immunized *cynomolgus* monkeys to obtain information of the immune response relevant to the safety of the S2 vaccine at an early stage. Toward this end, instead of using the lymph tissues or organs, in this study, we took an alternative way, i.e., using the peripheral blood to isolate lymphocytes for transcriptomic analysis. We did not examine the lymph nodes for the innate immune response due to our concern that the fixation of animals and the collection of blood may lead to unexpected abortion, and as a consequence, it may interfere with our evaluations on the safety of the S2 vaccine, since abortion is an important parameter in our evaluation in this study. The use of the peripheral blood for the isolation of lymphocytes for transcriptomic analysis is not new, as the use of the peripheral blood from pigs (30), dogs (31), Chinese geese (32), and humans (33–35), including patients with COVID-19 (36), have been well-documented.

Previously, comparative genomic analysis between S2 and the virulent strain 1,330 was reported, and 59 different open reading frames (ORFs) between these two strains were identified, several of which were demonstrated to be related to the virulence of *Brucella* spp., such as outer membrane autotransport and eryD (16). To further uncover the mechanism underlying the virulence attenuation of S2, our colleagues recently characterized the transcriptional profile of S2 and *B. suis* 1,330 infected murine macrophages by the transcriptome analysis, showing that the expression of 440 genes was significantly enriched, involving the innate immune response, phagocytosis, recognition, and inflammatory response, as well as the *Staphylococcus aureus* infection pathway and the NF-kappa B signaling pathway (28). In addition, our findings indicated that the DEGs related to the S2 immunization/infection were different between murine macrophages and monkeys (28). In order to further ascertain the host cellular response and genetic factors associated with S2 vaccination in mammals, in this study, we performed a comparative transcriptome analysis on blood cells collected from S2-immunized *cynomolgus* monkeys. We demonstrated that S2 vaccination of monkeys resulted in altered levels of the expression of 663 genes relevant to various MFs and biological functions of the host organism, including actin cytoskeleton regulation and binding, defense response, 2'-5'-oligoadenylate synthetase activity, dsRNA binding, the type-I interferon signaling pathway, and the chemokine signaling pathway. These results enhanced our understanding of the immune response of the mammalian species upon S2 vaccination as well as the mechanism of virulence attenuation of the S2 vaccine strain.

The data reported in the present study shed light on the safety of the S2 vaccine. Yet, there are still some limitations in our study. First, only limited numbers of *cynomolgus* monkeys were used, resulting in insufficient statistical analyses and comparisons. Second, the blood of pregnant *cynomolgus* monkeys was not collected and tested; thus, we might have missed some important information about the transcriptomic profile of the pregnant *cynomolgus* monkeys. Third, we did not measure the parameters relevant to the host immune response, such as the humoral immunity and the cell-mediated immune response. Fourth, RT-PCR detecting *Brucella* in the collected peripheral blood was not performed. These outcomes may suggest that only a partial situation of the safety of the S2 vaccine was truly reflected. Despite these limitations, we believe that our results would enhance the understanding of the safety of the S2 vaccine. Further studies with improved experimental design and larger sample size are warranted to further verify the reported results.

## Conclusion

This study provides evidence on the safety of the *Brucella* S2 vaccine strain on *cynomolgus* monkeys, enhancing the understanding of the pathogenicity of S2 in human. The work offers insights into the molecular basis of the host gene regulation in response to S2 vaccination.

## Data Availability Statement

The datasets presented in this study can be found in online repositories.The names of the repository/repositories and accession number(s) can be found below: NCBI Gene Expression Omnibus, Accession No: GSE165283.

## Ethics Statement

The animal study was reviewed and approved by Animal Ethics Committee of China Institute of Veterinary Drug Control, China.

## Author Contributions

LZ, JD, and YQ conceived and designed the experiments. SS, HJ, QL, and YL performed the experiments, analyzed the data, and prepared figures and tables. QG, YF, XP, GX, QS, and XF performed the experiments and analyzed the data. All authors reviewed the drafts of the paper and approved the final draft.

## Conflict of Interest

The authors declare that the research was conducted in the absence of any commercial or financial relationships that could be construed as a potential conflict of interest.
